# Resection of retrohepatic inferior vena cava without reconstruction in ex vivo liver resection and autotransplantation: a retrospective study

**DOI:** 10.1186/s12893-020-00720-z

**Published:** 2020-03-24

**Authors:** Xianwei Yang, Tao Wang, Junjie Kong, Bin Huang, Wentao Wang

**Affiliations:** 1 Department of Liver Surgery & Liver Transplantation Center, West China Hospital of Sichuan University, 37 Guoxue Road, Chengdu, 610041 P. R. China; 2 Department of Vascular Surgery, West China Hospital of Sichuan University, Chengdu, P. R. China

**Keywords:** Hepatic alveolar echinococcosis, Inferior vena cava, Liver autotransplantation, Reconstruction

## Abstract

**Background:**

Retrohepatic inferior vena cava (RIVC) resection without reconstruction in ex vivo liver resection and autotransplantation (ERAT) for advanced alveolar echinococcosis (HAE) is unclear.

**Methods:**

This is a retrospective study of consecutive patients referred to our hospital from 2014 to 2018. Depending on the presence of a rich collateral circulation and stable blood volume in ERAT, patients did not rebuild the RIVC. Then, patients were selected some appropriate revascularization techniques for the hepatic and renal veins. Finally, all ERAT procedures were completed, and short- and long-term outcomes were observed.

**Results:**

Five advanced HAE patients underwent ERAT without RIVC reconstruction. One patient died of circulatory failure 1 day after surgery. Another four patients, with a median follow-up duration of 18 months (range, 10–25 months), demonstrated normal liver and kidney function, no thrombosis and no HAE recurrence.

**Conclusions:**

Through the long-term results of ERAT, the pros and cons of not reconstructing the RIVC need to be re-examined. In cases with a rich collateral circulation, the RIVC cannot be reconstructed. However, in cases requiring the resection of multiple organs, RIVC without reconstruction was prudential.

## Background

Intraabdominal inferior vena cava (IVC) resection is a very challenging task. When the IVC is invaded by primary or secondary lesions of the liver, the hepatic tumors and the retrohepatic inferior vena cava (RIVC) should be treated simultaneously [1, 2]. Correspondingly, protecting blood flow in the portal system, kidneys and lower limbs is also challenging. Ex vivo liver resection and autotransplantation (ERAT) has become a hot topic in recent years [3], especially in the study of benign liver diseases. An increasing number of new ideas support the superior long-term efficacy of ERAT in treating hepatic alveolar echinococcosis (HAE) or benign liver tumors compared to that of allogeneic liver transplantation [4, 5]. In patients with HAE with partial or complete occlusion of the RIVC before surgery, if the imaging evaluation shows a rich collateral circulation, RIVC reconstruction may not be performed according to the actual situation [2]. However, there have been few relevant previous studies and no long-term outcome studies, and most related studies have examined primary IVC tumors [6, 7]. This study analyzed the technical features and short-term and long-term results of RIVC resection without reconstruction in ERAT in a 2-year follow-up study at our center.

## Methods

From February 2014 to August 2018, a total of 280 HAE patients underwent surgical treatment at our Medical Center, West China Hospital of Sichuan University, and the medical records were retained. Seventy-seven patients (27.5%) with advanced HAE underwent ERAT, and the RIVC was not reconstructed in five of them (6.5%). Table 1 showed the clinical characteristics and imaging findings of these 5 patients.

**Table 1:** The clinical characteristics and imaging findings of the 5 patients

Case	Age/Sex	BMI, kg/m^2^	Chief complaint	PNM stage	Diameter, cm	Pre-ERAT Treatment*	Estimated RLV (mL)	Autograft mass (g)	Follow up, months	Current status
1	32/M	17.7	Upper abdominal pain, 2 years	P4N1M0	14.1	PTCD	600	565	25	Alive
2	41/F	21.5	Upper abdominal pain, 4 months	P4N1M0	16.5	Hepatectomy	900	880	24	Alive
3	22/F	20.9	Liver lesion growth, 1 year	P4N1M0	9.2	Hepatectomy	450	380	13	Alive
4	44/F	18.8	Liver mass, 12 years; jaundice, 2 months	P4N1M1	14.0	PTCD	750	700	–	Dead
5	26/F	24.2	Liver lesion growth, 1 year	P4N1M1	11.2	No	750	730	10	Alive

*BMI* Body mass index, *PNM stage* the classifications P (parasitic in the liver), N (extension to neighboring organs), and M (distant metastasis) were developed by the European Echinococcosis Registry Network of the WHO Informal Working Group on Echinococcosis, *ERAT* ex vivo liver resection and autotransplantation, *PTCD* percutaneous transhepatic cholangial drainage, *RLV* remnant liver volume (preoperative measurement by CT images). Patient 4 died 1 day after surgery, so there was no follow up. "*" refered to the pre-ERAT treatment measures, not the current admission (ERAT), and the interval was at least 2 months. Gender, age and clinical data of the patients were available

The study was approved by the Ethics Committee of West China Hospital of Sichuan University (No. 2017–38) and conducted in accordance with the Declaration of Helsinki. All patients and their families signed informed consent and surgical consent before surgery. Calculation of the mean, median, range, and frequency was performed with statistical Excel 2016 software (Microsoft, Redmond, WA, USA).

### Surgical technique

**Fig. 1:**
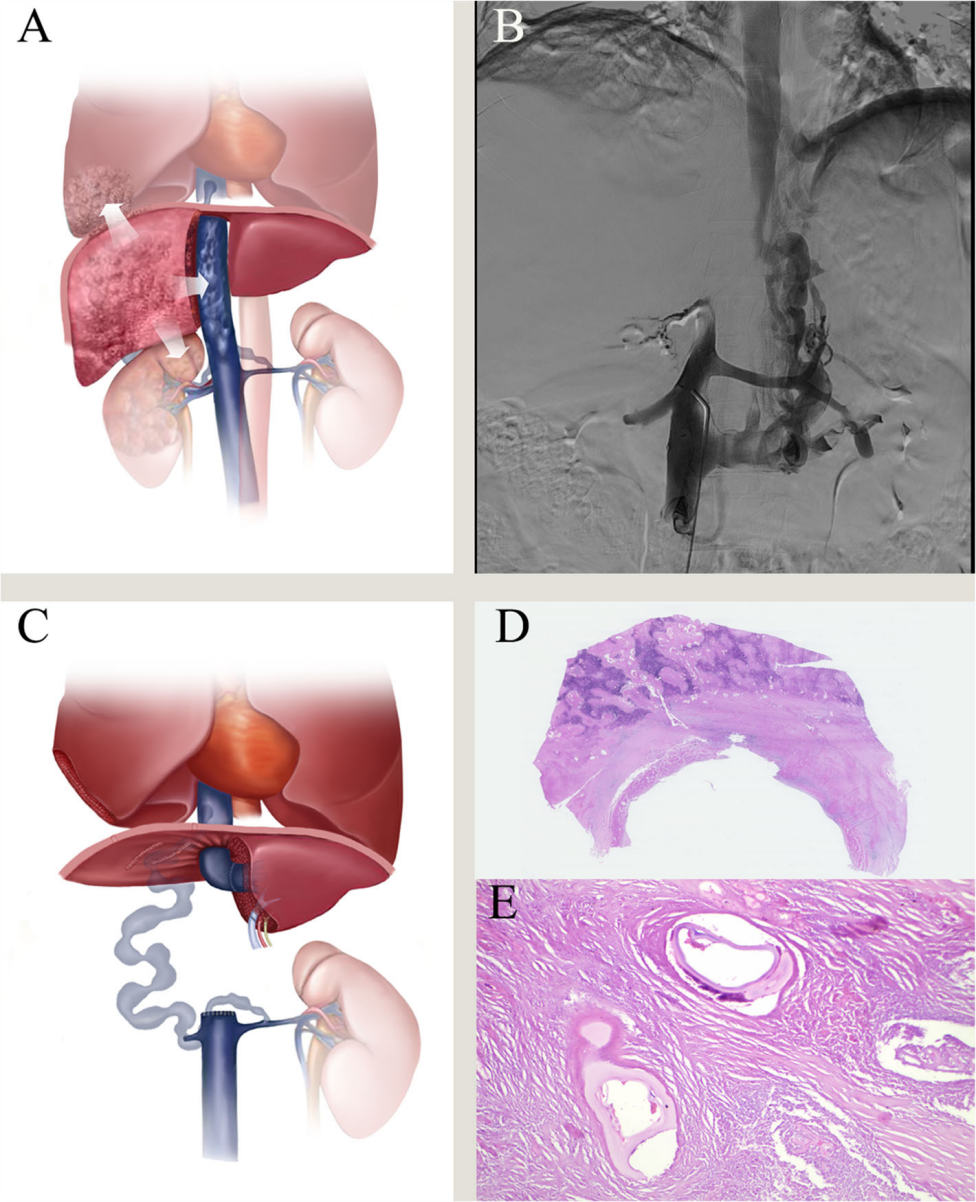
The main techniques for retrohepatic inferior vena cava (RIVC) resection without reconstruction in ex vivo liver resection and autotransplantation (ERAT). **a** preoperative simulation of the extent of hepatic alveolar echinococcosis, including the right lower lung lobe and right kidney. **b** preoperative inferior vena cava angiography shows the rich collateral circulation and right renal vein, retrohepatic inferior vena cava occlusion. **c** diagram of reconstruction of patient 4 after liver autotransplantation. **d** histological section of the all layers of RIVC showing a granulomatous inflammation with a necrotic center induced by Echinococcus multilocularis. Hematoxylin–eosin staining, × 0.37. **e** remnants of laminated layer of E. multilocularis. Hematoxylin–eosin staining, × 400. Figure **a** and **c** were draft completed by Xianwei Yang and the final draft completed in Adobe Photoshop CS6 (Serial number: 1330–1152–1635-8311-0579-3839). These figure 1**a**-**e** belongs to the authors, and editing is allowed

The surgical indications for ERAT were consistent with our previous reports [2], and five patients first met this criterion. Based on the location of the HAE lesions, residual liver function, kidney function, and the assessment of extrahepatic organs (e.g., lungs and brain, Fig. 1a), after a multidisciplinary team discussion, we prepared a plan preoperatively for not reconstructing the RIVC in these 5 patients. Preoperative IVC angiography and ultrasound were used to assess the collateral circulation (Fig. 1b). Artificial blood vessels (InterGard, InterVascular SAS, Inc., La Ciotat, France) were prepared for these patients if IVC vascular replacement was needed. The Mercedes incision was used to enter the abdominal cavity, fully exposing the liver, hilar structure and IVC. Due to the large size of the HAE lesions, it was often necessary to remove some of the diaphragm and empty the gastrointestinal tract. The treatment of the hepatic artery, portal vein, bile duct and other vessels has been detailed in a previous report [2, 8]. In five patients, ex vivo liver resection and back-table reshaping was performed to obtain healthy liver grafts. Then, the patients entered the anhepatic phase.

In the anhepatic phase, a temporary portocaval shunt was used in 3 patients because these patients showed slight intestinal congestion. The ex vivo liver resection was simultaneously performed by another group of surgeons. The liver was perfused with 4–8 L of 0–4 °C HTK solution (histidine-tryptophan-ketoglutarate, Custodiol®, Dr. Franz Kohler Chemie, Germany) via the PV while cooled in ice bath. Parenchymal transection was performed with a minimum 1.0-cm lesion-free margin using the Cavi-Pulse Ultrasonic Surgical Aspirator (CUSA, Valleylab, Boulder, CO, USA). In patients 1 and 2, temporary artificial blood vessels were used instead of the RIVC. Finally, in patient 1, only the artificial blood vessels in the left hepatic vein and the suprahepatic IVC junction were retained, while in patient 2, the artificial vessels were all discarded after the suprarenal IVC was closed. In patient 4, the shaped suprarenal IVC and the end of the left renal vein were directly anastomosed (Fig. 1c). Because HAE invaded the right kidney in patient 4, it could not be preserved and needed to be removed. Ligation of the right renal vein was possible due to the existence of a rich collateral circulation through the left renal, adrenal and gonadal veins. In all patients, the hemodynamics and urine volume remained stable, without intestinal congestion after RIVC clamping (duration, over 30 min) or end-to-end anastomosis, particularly when the patients had thriving ascending lumbar veins, an adequate hemiazygos, or large portosystemic collaterals. See Figure S1 for details of the other operations characteristics of the 5 patients.

In patients 1 and 3, the suprahepatic IVC was anastomosed after the great saphenous vein was used to shape the left hepatic vein (enlarged outflow tract). Anastomosis was carried out using running sutures of 5–0 Prolene. The left hepatic artery was reconstructed in an end-to-end manner to a size-matched branch of the hepatic artery, and duct-to-duct biliary anastomosis was performed in all patients. The anastomosis of these vessels was completed after the intraoperative ultrasound monitoring of hemodynamics was normal. After repeated examination of the abdominal cavity, two drainage tubes were placed, and the abdominal cavity was closed satisfactorily. Immediately after the infected IVC and liver lesions were removed, they were placed in 10% formaldehyde, routinely dehydrated, embedded in paraffin, sliced into 4 μm sections, and hematoxylin and eosin (HE) was performed. The diagnosis was based on the presence or absence of small particles of E. multilocularis (spems) outside the main lesion under the microscope.

### Postoperative management and follow-up observations

After the operation, the patient was admitted to the intensive care unit and returned to the general ward after recovering vital signs. No antirejection drugs or anticoagulant drugs were needed after the surgery. All patients were administered preferentially albendazole (15 mg/kg/day) routinely for 1 year after ERAT [9]. Albendazole was stopped if patients showed adverse events (including hypersensitivity, drug-induced liver injury, intolerance, or allergy), change the dosage form, or take the medicine 2 months after the side effect disappears. Short-term (< 90 days) and long-term postoperative complications were identified and treated. The patients were followed every 3–6 months after discharge.

## Results

We retrospectively collected 5 HAE patients (4 women, 1 man) who underwent ERAT without RIVC reconstruction (Table 1). The mean age of these patients was 33 years (range, 22–44 years). The chief complaint was upper abdominal pain, which was observed in 2 patients. Among the patients, patients 1 and 4 showed obstructive jaundice due to lesion compression, and they underwent percutaneous transhepatic cholangial drainage (PTCD) to reduce the total bilirubin level before admission.

The median operative duration was 859 min (range, 600–1290 min), and the anhepatic period was 326 min (range, 197–440 min). The median blood loss was 4140 ml, and the range was 2200–800 ml. Postoperative pathology was confirmed as HAE, and RIVC was violated in the whole layer (Fig. 1d, e). The average postoperative hospital stay was 21 days (range, 1–47 days). Short-term complications included one case of mortality (patient 4) due to circulatory failure 1 day after surgery and two cases of bile leakage (patients 3 and 5), which disappeared after 14 days abdominal drainage. Hepatic vein stenosis occurred in patient 2 at 6 months after the surgery and was relieved after stent implantation. Patient 3 showed sacral effusion in the liver at 5 months after the surgery and finally underwent puncture under ultrasound guidance, which indicated biliary cysts. Postoperative IVC angiography showed that the suprarenal IVC was completely blocked and multiple collateral circulation routes had been established. The intra- and postoperative characteristics and complications are summarized in Table 2. None of the 4 surviving patients showed HAE recurrence and were alive with a patent collateral circulation after a median follow-up period of 18 (range, 10–25 years) months.

**Table 2:** Intra- and postoperative characteristics and complications

Case	OP time (min)	Anhepatic phase (min)	Blood loss (ml)	Transfusion (RBC units)	RIVC length (cm)	RIVC circumference	Short-term complication	Long-term complication	Complication management
1	720	304	4000	20	6	360	–	–	–
2	905	428	2200	10	7	270	Abdominal cavity effusion	Hepatic vein stenosis, repeated ascites	Stent implantation
3	780	260	3000	6	8	360	Bile leakage	Bile cyst	Puncture drainage
4	1290	440	8000	28	8	360	Circulatory failure, death	–	–
5	600	197	3500	8	7	300	Bile duct stricture	–	ERCP, ENBD

*OP* operative, *RBC* red blood cell, *RIVC* retrohepatic inferior vena cava, *ERCP* endoscopic retrograde cholangiopancreatography, *ENBD* endoscopic nasal biliary drainage

## Discussion

Like IVC leiomyosarcoma, many benign or malignant lesions of the liver that invade the IVC are challenging to treat [7]. Echinococcosis is a benign parasitic disease that is mainly transmitted through the “fecal-mouth” route (digestive tracts). Because most of the epidemic areas are in high-altitude Tibetan areas (average elevation of 4200 m), medical resources are scarce, education levels are low, and many patients are in advanced stages of treatment, and HAE is more severe than cystic hydatid disease [10, 11]. HAE is a seriously neglected parasitic disease that is also known as worm cancer [11, 12]. When the hepatic echinococcosis lesion is huge, it often invades the inferior vena cava [13]. Unlike liver tumor biology (eg, primary liver cancer, hilar cholangiocarcinoma), HAE is a benign disease that is not in an irreparable state when it invades vital blood vessels and organs. Because the palliative treatment and /or anti-infective treatment (oral albendazole tablets) doesn’t achieve the desired results, surgery is the primary treatment for advanced HAE [2, 14].We describe 5 cases of HAE with a blocked RIVC treated with RIVC resection without reconstruction. Although the surgical technique might be a feasible and safe method for HAE patients with an adequate collateral circulation, the surgical outcome also deserves reflection. AE is close to the normal IVC outer membrane layer, mainly chronic granulomatous inflammation, accompanied by a large accumulation of lymphocyte, gradually manifested as chronic fibrosis, forming an inflammatory response zone. This is similar to liver AE lesions. For ERAT without reconstructing IVC, it is not necessary to accurately describe the inflammation of these lesions. We are more concerned about the degree of stenosis (perimeter) and length of invasion of the IVC. If the stenosis is lighter and shorter, this complicated operation is not necessary. Using local resection and reconstruction of IVC is sufficient [15].

For a long time, the RIVC has been considered to not require reconstruction if there is a rich collateral circulation [4, 7]. According to this view, we did not the reconstruct RIVC in 5 patients. Patient 2 developed postoperative hepatic vein stenosis, mainly due to anastomotic stenosis associated with the suprahepatic IVC; however, the death of patient 4 was considered to be closely related to the lack of RIVC reconstruction. Due to the large volume of blood loss during surgery, the blood volume was supplemented by multiple venous channels after surgery, but the circulation still could not compensate for the circulatory failure, eventually leading to death. At this time, through the bridge of the RIVC and the left renal vein, the collateral circulation and the blood flow in the lower limbs was insufficient for the heart. The response to angiotensin and antishock drugs was also insufficient. In addition, patient 4 underwent right lung lobectomy, right nephrectomy, and liver graft resection, which was another factor contributing to the slow postoperative recovery. Therefore, we believe that when RIVC resection is combined with multiple organ resection, the operative duration is relatively long, and the blood loss volume is relatively large, even if there is a sufficient collateral circulation, the IVC should be reconstructed.

## Conclusions

This study illustrates the surgical technique and short- and long-term outcomes of RIVC resection without reconstruction due to complex hepatic lesions inducing a rich collateral circulation. Although this study was limited by the number of cases and the follow-up duration, we recommend this procedure for use in ERAT (liver autotransplantation), Budd-Chiari syndrome, and primary IVC tumors but recommend that the surgeon think twice before not rebuilding the IVC.

## Supplementary information

**Additional file 1: Figure S1.** The main preoperative assessment and surgical techniques for retrohepatic inferior vena cava (RIVC) resection without reconstruction in ex vivo liver resection and autotransplantation (ERAT). a. preoperative imaging assessment of the extent of hepatic echinococcosis. b. shows the IVC blocked in back-table preparation. c. the treatments of inferior vena cava in the anhepatic phase. d. after the completion of the pipeline’s reconstruction, the liver blood supply was good.

## Data Availability

The datasets used and/or analysed during the current study are available from the corresponding author on reasonable request.

## Abbreviations

ERAT: Ex vivo liver resection and autotransplantation
HAE: Hepatic alveolar echinococcosis
IVC: Intraabdominal inferior vena cava
RIVC: Retrohepatic inferior vena cava

## References

[1] Bi Y, Chen H, Ding P (2018). Comparison of retrievable stents and permanent stents for Budd-Chiari syndrome due to obstructive inferior vena cava. J Gastroenterol Hepatol.

[2] Yang X, Qiu Y, Huang B (2018). Novel techniques and preliminary results of ex vivo liver resection and autotransplantation for end-stage hepatic alveolar echinococcosis: a study of 31 cases. Am J Transplant.

[3] Beldi G, Vuitton D, Lachenmayer A (2019). Is ex vivo liver resection and autotransplantation a valid alternative treatment for end-stage hepatic alveolar echinococcosis in Europe?. J Hepatol.

[4] Aji T, Dong JH, Shao YM (2018). Ex vivo liver resection and autotransplantation as alternative to allotransplantation for end-stage hepatic alveolar echinococcosis. J Hepatol.

[5] Shen S, Kong J, Qiu Y (2019). Ex vivo liver resection and autotransplantation versus allotransplantation for end-stage hepatic alveolar echinococcosis. Int J Infect Dis.

[6] Yoon YI, Lee SG, Moon DB (2019). Surgical techniques and long-term outcomes of living-donor liver transplantation with inferior vena cava replacement using Atriocaval synthetic interposition graft for Budd-Chiari syndrome. Ann Surg.

[7] Wachtel H, Jackson BM, Bartlett EK (2015). Resection of primary leiomyosarcoma of the inferior vena cava (IVC) with reconstruction: a case series and review of the literature. J Surg Oncol.

[8] Qiu Y, Yang X, Shen S (2019). Vascular infiltration-based surgical planning in treating end-stage hepatic alveolar echinococcosis with ex vivo liver resection and autotransplantation. Surgery.

[9] Kern P, Wen H, Sato N (2006). WHO classification of alveolar echinococcosis: principles and application. Parasitol Int.

[10] Botrugno I, Gruttadauria S, Li PS (2010). Complex hydatid cysts of the liver: a single center's evolving approach to surgical treatment. Am Surg.

[11] Wen H, Vuitton L, Tuxun T, et al. Echinococcosis: Advances in the 21st Century. Clin Microbiol Rev. 2019;32(2). 10.1128/CMR.00075-18. 10.1128/CMR.00075-18 PMC6431127 30760475

[12] Yang X, Qiu Y, Wang W (2017). Risk factors and a simple model for predicting bile leakage after radical hepatectomy in patients with hepatic alveolar echinococcosis. Medicine (Baltimore).

[13] Jaiswal P, Jaiswal R, Attar BM (2018). Hepatobiliary and pancreatic: massive hepatic cystic echinococcosis compressing inferior vena cava. J Gastroenterol Hepatol.

[14] Du C, Liu Z, Yang X (2016). Hepatectomy for patients with alveolar echinococcosis: long-term follow-up observations of 144 cases. Int J Surg.

[15] Shen S, Kong J, Zhao J (2019). Outcomes of different surgical resection techniques for end-stage hepatic alveolar echinococcosis with inferior vena cava invasion. HPB (Oxford).

