# Supplementary figures and images for: Resection of retrohepatic inferior vena cava without reconstruction in ex vivo liver resection and autotransplantation: a retrospective study
Source: BMC Surg. 2020 Mar 24;20:56. doi: 10.1186/s12893-020-00720-z (PMC7092598; doi:10.1186/s12893-020-00720-z)

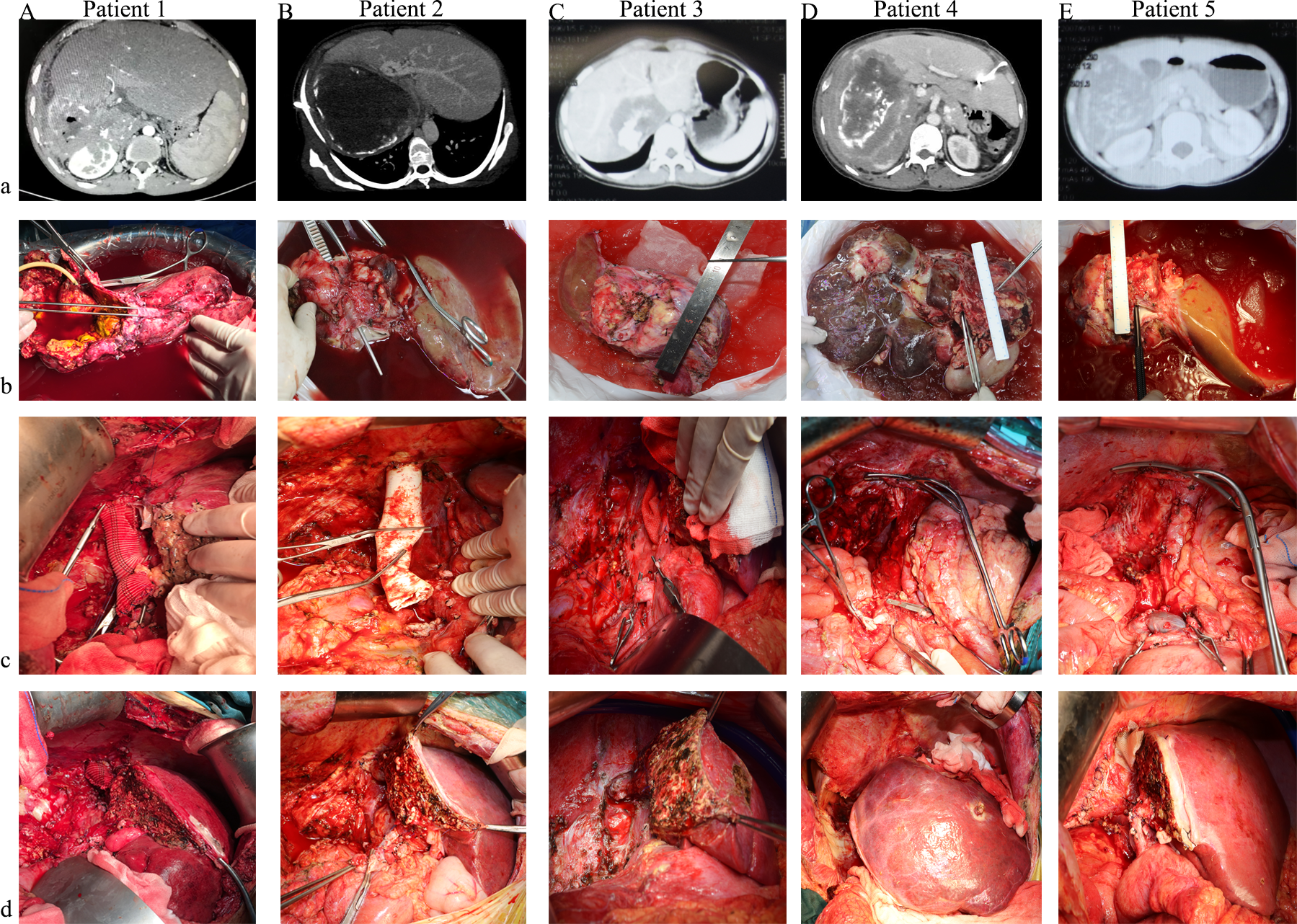

Supplement: Supplementary file 1 — Additional file 1: Figure S1. The main preoperative assessment and surgical techniques for retrohepatic inferior vena cava (RIVC) resection without reconstruction in ex vivo liver resection and autotransplantation (ERAT). a. preoperative imaging assessment of the extent of hepatic echinococcosis. b. shows the IVC blocked in back-table preparation. c. the treatments of inferior vena cava in the anhepatic phase. d. after the completion of the pipeline’s reconstruction, the liver blood supply was good. [file 12893_2020_720_MOESM1_ESM.tif]
